# Multiple thrombi mimicking metastases in the right atrium of patients with non-Hodgkin’s lymphoma diagnosed by multimodal cardiac imaging: one case report

**DOI:** 10.1186/s13019-024-02650-w

**Published:** 2024-04-01

**Authors:** Zhiqiang Hu, Shuai Yuan, Yun Mou

**Affiliations:** Department of Echocardiography and Vascular Ultrasound Center, The First Affiliated Hospital, School of Medicine, Zhejiang University, #79 Qingchun Road, Hangzhou, Zhejiang Province 310003 P.R. China

**Keywords:** Lymphoma, Right atrium mass, thrombus, Multimodal cardiac imaging

## Abstract

**Background:**

Right-side heart mass can be found incidentally on routine transthoracic echocardiography (TTE). Accurate diagnosis of cardiac mass often requires more than one imaging method. We present a mid-age woman with non-Hodgkin lymphoma who was found to have multiple right atrial masses mimicking metastases on routine TTE, which were finally diagnosed as thrombi by multimodal cardiac imaging.

**Case presentation:**

A 52-year-old woman was diagnosed with primary mediastinal diffuse large B cell lymphoma (DLBCL) almost six months prior. The TTE revealed multiple masses in the right atrium with normal cardiac function when she was being evaluated for the next chemotherapy. On arrival, she was hemodynamically stable and asymptomatic. Physical examination was no remarkable. Laboratory findings showed leukocytosis of 17,900 cells/mm3, hemoglobin of 7.5 mg/dL, and a normal D-dimer level. The suspicious diagnosis of right atrial metastasis was made by TEE. However, the diagnosis of right atrial thrombi was made by contrast CMR. Finally, the 18 F-FDG PET-CT demonstrated no metabolic activity in the right atrium, which further supported the diagnosis of thrombi. Eventually, the masses were removed by cardiopulmonary bypass thoracotomy because of a high risk of pulmonary embolism. Histopathology confirmed the diagnosis of thrombi.

**Conclusions:**

This case highlights the importance of multimodality cardiac imaging in the appropriate diagnosis of a RA masses in patient of lymphoma. Diagnosis of RA masses can be made using multimodal cardiac imaging like TTE, TEE and CMR, even PET. Echocardiography is the most commonly used on multimodal imaging in cardiac thrombus. CMR has high specificity in differentiating a tumor from thrombus, while 18 F-FDG PET has good sensitivity to determine the nature of the masses.

## Background

Right-side heart thrombus can be found incidentally on routine TTE. Its early and accurate diagnosis is beneficial to clinical management. Accurate diagnosis of cardiac thrombus often requires more than one imaging method. Echocardiography is the most commonly used diagnostic method, and CMR is the gold standard for noninvasive diagnosis of cardiac thrombus. PET-CT has good sensitivity and specificity in the diagnosis of cardiac thrombus, and has become one of evaluation methods of cardiac thrombus [1]. We present a case of RA thrombi in a patient with non-Hodgkin lymphoma (NHL), in which diagnosis was confirmed with the aid of multimodal cardiac imaging.

## Case presentation

A 52-year-old woman was diagnosed with primary mediastinal of differentiation CD20 (+), CD79a (+), Bcl-2 (+), Bcl-6 (partial+), MUM1 (partial+) diffuse large B cell lymphoma (DLBCL) almost six months prior. She was found to have stage IV disease with diffuse involvement of the thymus, lymph nodes and bone marrow. A power-injectable port was inserted and five cycles of R-DAEPOCH chemotherapy (rituximab, etoposide, epirubicin, vindesine, dexamethasone, cyclophosphamide) have performed. During chemotherapy cycles, the patients have no special discomfort symptoms. The transthoracic echocardiography (TTE) revealed multiple masses in the right atrium with normal cardiac function when she was being evaluated for the next chemotherapy. She was then sent to the in-patient department for further evaluation.

**Fig. 1:**
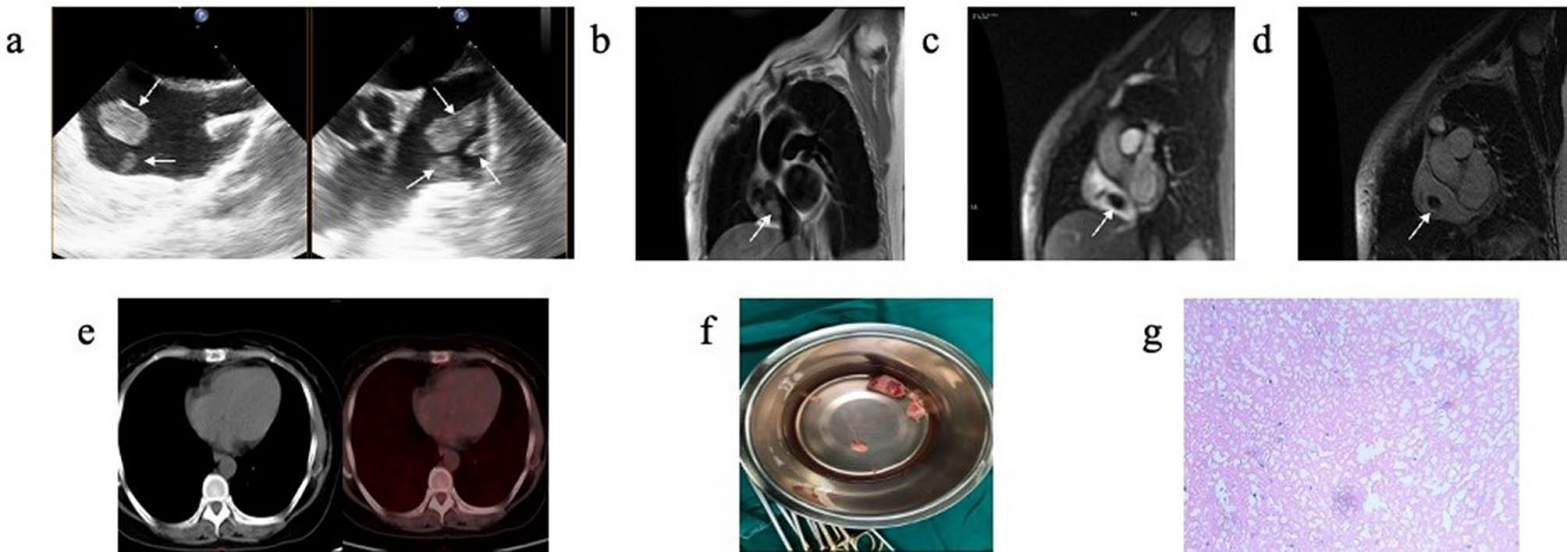
(**a**) TEE showed multiple oval homogenous masses attached to the atrial wall with thin sticks, swinging with cardiac cycle, and no obvious thickening of the right atrial wall. (**b**) CMR showed isointense signal on black blood T2-weighted imaging. (**c**) CMR gadolinium contrast injection showed no enhancement of the RA masses in early priod. (**d**) CMR gadolinium contrast injection showed no enhancement of the RA masses in delayed priod. (**e**) PET-CT showed no abnormal elevated FDG metabolism in the RA. (**f**) During the surgery, multiple occupying lesions were visible in the right atrium, varying in size and brittle as pearls. The largest one was about 3*2 cm in size, and no obvious abnormalities were observed in the tricuspid valve ring and right atrial wall. (**g**) The histopathological (200×) after surgery confirmed that the right atrial masses were thromboid tissue with calcium deposition

On arrival, she was hemodynamically stable and asymptomatic. Physical examination was no remarkable. Laboratory findings showed leukocytosis of 17,900 cells/mm3, hemoglobin of 7.5 mg/dL, and a normal D-dimer level. To further clarify the diagnosis, TEE showed multiple oval homogenous masses attached to the atrial wall with thin sticks, swinging with cardiac cycle, and no obvious thickening of the right atrial wall. (Fig. 1a). Because there was a question of differentiating between metastatic lesions vs. thrombi, CMR was also obtained which showed isointense signal on black blood T2-weighted image (Fig. 1b) with no enhancement of the RA masses on gadolinium contrast injection in early (Fig. 1c) and delayed periods (Fig. 1d), supporting the diagnosis of thrombi. In addition, PET-CT showed no abnormal elevated FDG metabolism in the right atrium (Fig. 1e). The patient finally underwent surgical treatment because of high risk of pulmonary embolism. Multiple occupying lesions were visible in the right atrium (Fig. 1f) during the surgery, and the histopathological confirmed that the right atrial masses were thrombi (Fig. 1g).

## Discussion

When a mass is found in the right atrium, it should be differentiated from cardiac or non-cardiac tumor. Among non-cardiac tumor, right atrium thrombus is the most common mass. Right atrium thrombus can be classified into “emboli in transit” and thrombus in situ, which was often associated with medical devices [2]. A thrombus in situ often appears smaller, less mobile, homogenous, and demonstrate no enhancement due to their avascularity after contrast injection.

Right atrium mass can be detected by TTE initially. However, there are several limitations, including operator dependence, a restricted field of view in TTE. When there is diagnostic doubt, TEE can provide some additional diagnostic information-but TEE is an invasive test. Furthermore, CT or CMR [3], and PET-CT [4] often become the methods for further differential examination. CMR imaging has become the gold standard techniques in the evaluation of cardiac masses, which can be used to evaluate the signal characteristics and morphological characteristics of cardiac mass, and help to determine the nature of mass lesions [5]. MR imaging characteristics can be used to predict the likely malignancy of a cardiac mass. Some studies have shown that the accuracy of MR in differentiating benign and malignant cardiac tumors may be more than 90% [3, 6]. However, the sensitivity of cardiac magnetic resonance imaging is insufficient, and PET just compensates for the sensitivity of cardiac magnetic resonance imaging, while cardiac magnetic resonance imaging also compensates for its poor specificity. Therefore, some studies suggested combining both methods for a more accurate diagnosis of cardiac masses [7].

Unfortunately, there is no noninvasive imaging modality determining malignancy of cardiac tumors with sufficient accuracy. Therefore, more imaging methods are needed to complement each other to provide better treatment options for patients. The cardiac masses in the patient mentioned here were detected by a routine TTE during chemotherapy for lymphoma, which were firstly suspected the possibility of metastases because of a history of malignancy in this patient. In order to further understand the characteristics of the cardiac masses, TEE was performed for the patient, and the masses were found with regular shape and attached to the right atrial wall with high mobility, but the nature of the masses could not be determined. Furthermore, CMR and PET-CT showed no blood flow in the masses and no obvious abnormal FDG metabolism in the right atrium, and finally determined that the masses in the right atrial were thrombi.

## Conclusions

This case highlights the importance of multimodality cardiac imaging in the appropriate diagnosis of a RA masses in patient of lymphoma. Diagnosis of RA masses can be made using multimodal cardiac imaging like TTE, TEE and CMR, even PET. Echocardiography is the most commonly used on multimodal imaging in cardiac thrombi. CMR has high specificity in differentiating a tumor from thrombi, while PET has good sensitivity to determine the nature of the masses.

## Data Availability

All data generated or analysed during this study are included in this published article.

## Abbreviations

TTE: transthoracic echocardiography
CMR: cardiac magnetic resonance
PET: positron emission tomography
FDG: fluorodeoxyglucose
NHL: non-Hodgkin lymphoma
RA: right atrium

## References

[1] Rahbar K (2012). Differentiation of malignant and benign cardiac tumors using 18F-FDG PET/CT. J Nucl Med.

[2] Goh FQ (2022). Clinical characteristics, treatment and long-term outcomes of patients with right-sided cardiac thrombus. Hellenic J Cardiol.

[3] Pazos-López P (2014). Value of CMR for the Differential diagnosis of Cardiac masses. JACC: Cardiovasc Imaging.

[4] Rinuncini M (2016). Differentiation of cardiac thrombus from cardiac tumor combining cardiac MRI and 18F-FDG-PET/CT imaging. Int J Cardiol.

[5] Motwani M (2013). MR imaging of cardiac tumors and masses: a review of methods and clinical applications. Radiology.

[6] Hoffmann U (2003). Usefulness of magnetic resonance imaging of cardiac and paracardiac masses. Am J Cardiol.

[7] Mikail N (2022). Diagnosis and staging of cardiac masses: additional value of CMR with (18)F-FDG-PET compared to CMR with CECT. Eur J Nucl Med Mol Imaging.

